# Supplementary material for: Laser Surface Microstructuring of a Bio-Resorbable Polymer to Anchor Stem Cells, Control Adipocyte Morphology, and Promote Osteogenesis
Source: Polymers (Basel). 2018 Dec 3;10(12):1337. doi: 10.3390/polym10121337 (PMC6401824; doi:10.3390/polym10121337)
Supplement: Supplementary file 1 [file polymers-10-01337-s001.pdf]

Supplementary material

Effect of surface topography on undifferentiated MSCs

Results:

Figure S1 shows the relative cell growth on non-modified PLLA surfaces (FLAT PLLA) as a function of the culture time compared to cell growth on glass coverslips at 1 day of incubation. Although MSC growth was slower on this material than on glass coverslips, there was noticeable cell growth, modest during the first week and then increased after 14 days. This is similar to other reported findings, for example, Prosecká and colleagues cultured MSC cells on PCL/PVA nanofibers and displayed a similar growth pattern, with relatively slow growth during the first week. In this case, they analyzed the effects of HA deposition, which caused a marked increase in cell viability, nevertheless, in each condition, growth at day 7 was similar to day 1, and only at day 14 significant cell growth was observed (Prosecká et al., J Biomed Biotechnol, 2012).

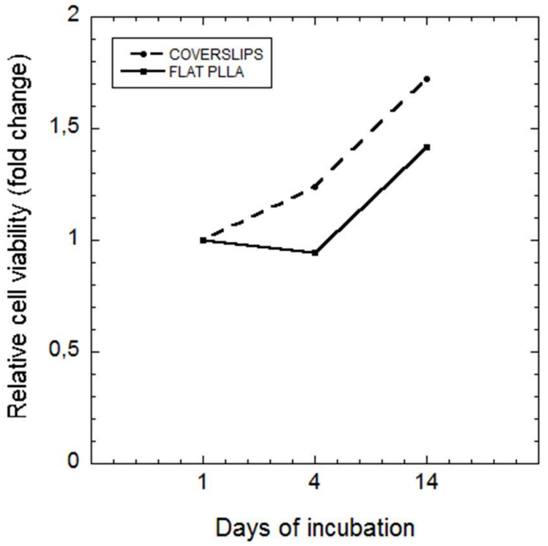

Figure S1. MSC proliferation on non-patterned PLLA (FLAT PLLA) normalized to cell growth on glass coverslips after 1 day in culture and compared to cell proliferation on glass coverslips at different days in culture.

Alignment of cell nucleus was quantified by ImageJ, using images of 5 different areas for each type of surface and incubation period. Each cell nucleus was marked and an ellipse was fitted to it to measure its orientation angle as the long axis vector. Figure S2 shows the polar graphs representing the angle at which each cell nuclei were oriented on every surface (measured by ImageJ) after 1 and 14 days in culture. All these graphs show that cell guidance clearly affected cell nuclei orientation when groove-patterned PLLA surfaces are considered compared to flat and rough PLLA surfaces. Polar graphs show cell nuclei with a wide range of orientation angles when cells grew on flat and rough PLLA surfaces, while cells growing on the groove-patterned PLLA surfaces showed nuclei with a noticeable trend to orientate along a preferred direction. Figure S3 shows the histograms representing how much the nuclei deviate from the main trend on GROOVES 1 and 2 surfaces at the same 2 different culture times. The histograms show that about 75% of the cell nuclei were oriented with deviation angles below 20º on the groove-patterned surface, when a groove configuration of 15 micrometres of inter-groove spacing was applied whereas approximately 60% of the nuclei were oriented in the same range of deviation angle, when grooves of 25 micrometres of inter-groove spacing were considered. Moreover, cell nuclei on grooves with the minor inter-groove spacing (s = 15 μm) were all aligned inside the laser-created channels (approximately (85 ± 5) %) and few nuclei were observed to be in the spacing between them ((15 ± 5) %). However, increasing inter-groove spacing (s = 25 μm), showed cell alignment, although in this case, many cell nuclei were found attached at the space between grooves ((46 ± 5) %).).

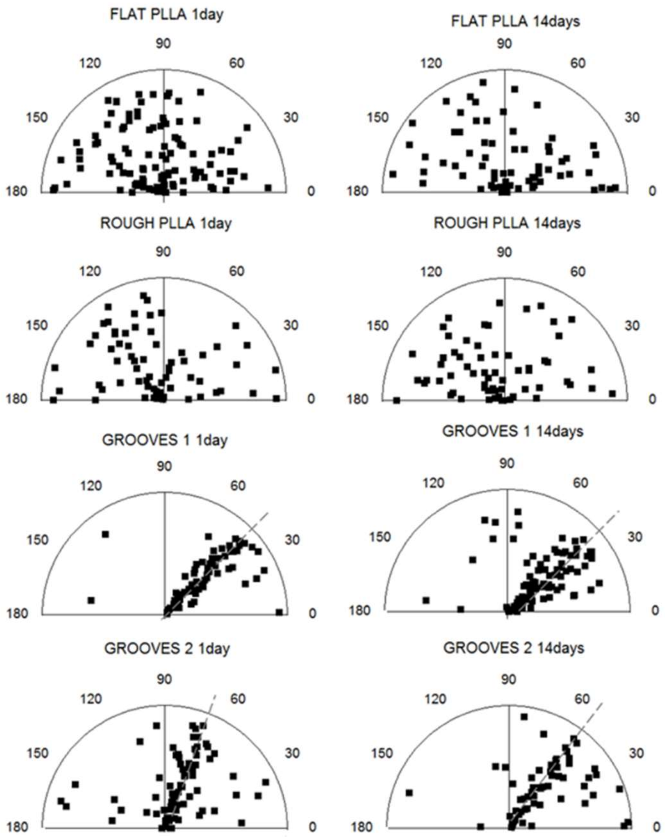

Figure S2. Polar graphs showing the angle of orientation of the cell nuclei on the 4 different PLLA surfaces (FLAT PLLA, ROUGH PLLA, GROOVES 1 and GROOVES 2) after 1 and 14 cultivation days. Each spot represents one cell nucleus. The dashed line highlights the main trend found on the groove-patterned surfaces.

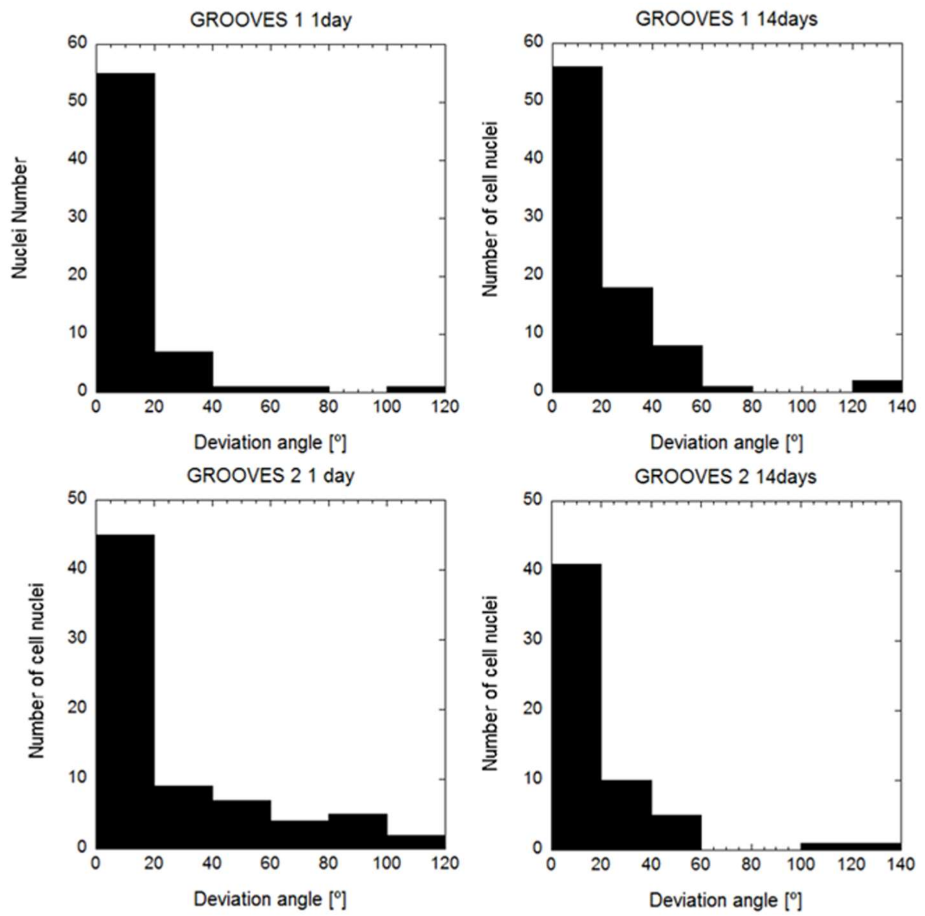

Figure S3. Histograms representing the deviation angle of cell nuclei respect to the trend angle on the patterned-PLLA surfaces (GROOVES 1 and GROOVES 2) after 1 and 14 cultivation days.
